# Supplementary material for: Classification for Penicillium expansum Spoilage and Defect in Apples by Electronic Nose Combined with Chemometrics
Source: Sensors (Basel). 2020 Apr 9;20(7):2130. doi: 10.3390/s20072130 (PMC7180459; doi:10.3390/s20072130)
Supplement: Supplementary file 1 [file sensors-20-02130-s001.pdf]

Table S1. PCA-DA results using all sensors data.

Calibration (72 samples)							Prediction (48 samples)						
	**Blank**	**1**	**2**	**3**	**—**	**Accuracy**		**Blank**	1	2	3	—	Accuracy
Blank	17	1	0	0	0		Blank	11	1	0	0	0	
1	0	17	1	0	0	95.83%	1	0	12	0	0	0	95.83%
2	0	0	17	1	0		2	0	0	11	1	0	
3	0	0	0	18	0		3	0	0	0	12	0	

Table S2. PCA-DA results using feature sensors data.

Calibration (72 samples)							Prediction (48 samples)						
	**Blank**	**1**	**2**	**3**	**—**	**Accuracy**		**Blank**	1	2	3	—	Accuracy
Blank	18	0	0	0	0		Blank	12	0	0	0	0	
1	0	18	0	0	0	97.22%	1	0	12	0	0	0	100%
2	0	1	16	1	0		2	0	0	12	0	0	
3	0	0	0	18	0		3	0	0	0	12	0	

Table S3. Classification results using PLS-DA algorithm using all sensor data.

Calibration (72 samples)							Prediction (48 samples)						
	**Blank**	**1**	**2**	**3**	**—**	**Accuracy**		**Blank**	1	2	3	—	Accuracy
Blank	18	0	0	0	0	100%	Blank	11	0	0	0	1	93.75%
1	0	18	0	0	0		1	0	11	0	0	1	

2	0	0	18	0	0		2	0	0	11	0	1
3	0	0	0	18	0		3	0	0	0	12	0

Table S4. Classification results using PLS-DA algorithm using feature sensors data

**Calibration (72 samples)**							**Prediction (48 samples)**						
	**Blank**	**1**	**2**	**3**	—	**Accuracy**		**Blank**	1	2	3	—	Accuracy
Blank	18	0	0	0	0		Blank	12	0	0	0	0	
1	0	16	0	0	2	95.83%	1	0	12	0	0	0	100%
2	0	0	17	0	1		2	0	0	12	0	0	
3	0	0	0	18	0		3	0	0	0	12	0	
